# The effectiveness of continuous quality improvement for developing professional practice and improving health care outcomes: a systematic review

**DOI:** 10.1186/s13012-020-0975-2

**Published:** 2020-04-19

**Authors:** James E. Hill, Anne-Marie Stephani, Paul Sapple, Andrew J. Clegg

**Affiliations:** 1 Faculty of Health and Wellbeing, University of Central Lancashire (UCLan), Preston, Lancashire PR1 2HE UK; 2 Warrington, UK

**Keywords:** Continuous quality improvement, Systematic review, Health care, Clinical process, Patient-based outcomes, RCTs

## Abstract

**Background:**

Efforts to improve the quality, safety, and efficiency of health care provision have often focused on changing approaches to the way services are organized and delivered. Continuous quality improvement (CQI), an approach used extensively in industrial and manufacturing sectors, has been used in the health sector. Despite the attention given to CQI, uncertainties remain as to its effectiveness given the complex and diverse nature of health systems. This review assesses the effectiveness of CQI across different health care settings, investigating the importance of different components of the approach.

**Methods:**

We searched 11 electronic databases: MEDLINE, CINAHL, EMBASE, AMED, Academic Search Complete, HMIC, Web of Science, PsycINFO, Cochrane Central Register of Controlled Trials, LISTA, and NHS EED to February 2019. Also, we searched reference lists of included studies and systematic reviews, as well as checking published protocols for linked papers. We selected randomized controlled trials (RCTs) within health care settings involving teams of health professionals, evaluating the effectiveness of CQI. Comparators included current usual practice or different strategies to manage organizational change. Outcomes were health care professional performance or patient outcomes. Studies were published in English.

**Results:**

Twenty-eight RCTs assessed the effectiveness of different approaches to CQI with a non-CQI comparator in various settings, with interventions differing in terms of the approaches used, their duration, meetings held, people involved, and training provided. All RCTs were considered at risk of bias, undermining their results. Findings suggested that the benefits of CQI compared to a non-CQI comparator on clinical process, patient, and other outcomes were limited, with less than half of RCTs showing any effect. Where benefits were evident, it was usually on clinical process measures, with the model used (i.e., Plan-Do-Study-Act, Model of Improvement), the meeting type (i.e., involving leaders discussing implementation) and their frequency (i.e., weekly) having an effect. None considered socio-economic health inequalities.

**Conclusions:**

Current evidence suggests the benefits of CQI in improving health care are uncertain, reflecting both the poor quality of evaluations and the complexities of health services themselves. Further mixed-methods evaluations are needed to understand how the health service can use this proven approach.

**Trial registration:**

Protocol registered on PROSPERO (CRD42018088309).

Contributions to the literature

- The paper presents the first systematic review of the effectiveness of continuous quality improvement (CQI) compared to non-CQI approaches on improving the quality, safety and efficiency in any health care sector;
- It assesses the importance of the health care setting, the CQI model used and key components of the different approaches used on changing clinical process and patient-based outcomes;
- The review examines the consideration given to socio-economic health inequalities in improving health care through CQI.

## Background

Improving the quality and safety of health care is a priority of governments, health care workers, and the public [1, 2], with efforts often focused on investment in changes to the way health care is organized and delivered (system-level quality improvements) [3, 4]. While there are many different approaches that may be taken, continuous quality improvement (CQI) has received considerable attention within health care [5] as a way to enhance the quality of care and reduce costs [6–9]. The use of CQI in health care has evolved since the 1990s, using quality control techniques and management theories employed in the industrial and manufacturing sectors [10–14]. In its earliest form, CQI was based on five main principles, specifically: a focus on organizational process and systems, rather than on individuals within the system; the use of statistically and methodologically robust structured problem-solving approaches; the use of multi-disciplinary team working; empowerment of employees to help identify problems and action improvement opportunities; and, a focus on “customers” (i.e., public) through an emphasis on creating the best possible patient experience and outcomes [13, 15, 16]. As the use of CQI has grown in health care, and new approaches to quality improvement have emerged from industry (e.g., total quality management), it is evident that the core features shared by the different methods have evolved [17–19]. A review of the characteristics of CQI in health care [20] identified three essential elements, which are systematic data-guided activities, iterative development and testing process, and designing with local conditions in mind [20]. Despite some uncertainty around the characteristics of CQI [21], several approaches encompass the fundamental principles and have been used in health, such as Lean Management, Six Sigma, Plan-Do-Study-Act (PDSA) cycles, and Root Cause Analysis [20].

Several systematic reviews have assessed the use of different approaches to help improve quality in health care, focusing on descriptions of the methods used and highlighting the differences in components included [22–32]. Those assessing CQI were in specific populations or clinical settings, considering their application [29], effectiveness [31], and the barriers and facilitators to the implementation of CQI [28, 30]. None compared the effectiveness of CQI across a range of health settings, assessed the benefits of specific components, or considered the actual impact of the factors that may influence the effects of CQI. Given these limitations, we systematically reviewed the evidence to assess the effectiveness of different approaches to CQI for developing professional practice and improving health care outcomes in any health care setting. We aimed to examine the impact of the various components encompassed in, and that affect the application of, the different approaches, which may act as facilitators or barriers to change. These components were based upon previously identified common features within CQI [20, 33] and criteria used to evaluate quality improvement interventions [34]. Also, we intended to consider the influence of socio-economic health inequalities on the effectiveness, and the implementation, of the approaches to CQI in improving health care. The importance of socio-economic inequalities in determining health, and the use of health and social care services, is widely recognized [35]. Increasingly, efforts are focusing on incorporating consideration of health inequalities in developing health and social care services to address the widening health gap [36].

## Methods

### Searches

Our systematic review followed recognized guidance and reporting standards (see Additional file 1 for PRISMA checklist) [37, 38], with the methods outlined in a research protocol registered on PROSPERO (CRD42018088309). We identified studies through searches of 11 electronic databases, specifically MEDLINE (via Ovid), CINAHL, EMBASE, AMED, Academic Search Complete, HMIC, Web of Science, PsycINFO, Cochrane Central Register of Controlled Trials, LISTA, and NHS EED (see Additional file 2 for example of search strategy). All databases were searched from their inception to 23 February 2019 and were limited to studies published in English. Additional references were identified through screening reference lists of all included studies and relevant systematic reviews. Linked companion publications were identified through checks of published study protocols.

### Study selection

Studies were eligible if they were randomized controlled trials (RCTs) within any health care setting involving teams of health professionals, evaluating the effectiveness of CQI (Table 1). Recognized features of CQI had to be present, including systematic data-guided activities, involvement of iterative development and testing, and a focus on a process or system rather than at an individual patient level [20]. Comparators could include different CQI strategies, current usual practice without an intervention to manage organizational change, or other non-CQI interventions to manage organizational change. Studies had to assess measures of health care professional performance (e.g., adherence to recommended practice or process of care) or patient outcomes (e.g., pain, health-related quality of life, mortality). Abstracts and conference proceedings were only considered if enough detail of their methodology and results were published. Study selection occurred through two stages. First, two reviewers independently screened the titles and abstracts of papers from the searches, using criteria specified prior to screening (Table 1). Discrepancies were discussed between reviewers, with arbitration by a third independent reviewer where required. Second, manuscripts of studies appearing to meet the selection criteria at title and abstract screening were retrieved. These were then screened using the same process as that for assessing titles and abstracts.

**Table 1:** Study selection criteria

Category	Inclusion criteria	Exclusion criteria
Participants	Teams of health professionals responsible for improving the health of their populations and/or providing patient care in any health care setting	Groups that do not include health professionals or that are conducted in a non-health care/non-public health setting or that only involve students.
Intervention	CQI that includes (i) use of measurement and data analysis to assess and review the effect of changes; (ii) review and analysis of a process or system used to deliver clinical care to identify sources of variation and areas for improvement; (iii) an iterative procedure within a continuous process; and (iv) a structured process improvement method or problem-solving approach that is used to plan and test changes to the work process.	Interventions targeting the improvement of administrative, management, or other processes not directly related to clinical care.
Comparison	Current usual practice (non-active control), different CQI strategies, or other non-CQI interventions to manage organizational change.	
Outcome	Any objective measure of health care professional performance (e.g., adherence to recommended practice or process of care) or patient outcome (e.g., pain, health-related quality of life, function, mortality).	
Study design	RCTs	

### Data extraction and study quality assessment

Data was extracted using a pre-piloted form by one reviewer and checked by a second reviewer. Disagreements were discussed between reviewers and, if consensus was not achieved, arbitration was carried out by a third reviewer. When further information was required, attempts were made to contact the authors for clarification. We extracted data on the characteristics of the CQI intervention that have previously been identified as important [20, 21, 38], including its scope; inclusion of factors considered key components of CQI, i.e., systematic data-guided activities, iterative development and testing process, and designing with local conditions in mind [20, 33]; and the use of important features of quality improvement in the implementation strategy (planned and actually implemented) [34]. Risk of bias was assessed using the Cochrane Collaboration tool by one reviewer, with decisions checked by a second reviewer [38]. Decisions on the key criteria of random sequence generation, allocation concealment, and blinding of patients and outcome assessment were also checked using a semi-automated process through RobotReviewer [39]. This involved uploading study text to, and checks being made against the criteria by, RobotReviewer. Where differences occurred, these were checked, justified and alterations made when required. Any disagreements were discussed, with arbitration by a third reviewer, if consensus was not reached.

### Data synthesis

The synthesis focused on those studies which compared a CQI intervention with a non-CQI intervention that was considered either current usual practice (i.e., without an intervention to manage organizational change) or another non-CQI intervention to manage change, allowing an assessment of the comparative benefits of the addition of CQI and limiting the effects of heterogeneity. Studies were synthesized through a narrative synthesis with a tabulation of results of included studies. Outcomes were separated into three groups, specifically clinical process outcomes, patient outcomes, and other outcomes. All outcomes were then categorized into five groups based on the ratio of outcomes demonstrating a statistically significant difference at the 5% significance level on the summary measures presented (i.e., risk ratios or mean difference with 95% confidence intervals) (Table 2). Differences were based on either the change from baseline to end of study (first data point after intervention) for CQI compared to that for control (difference within difference) or a comparison of CQI versus control at the end of the study with no statistically significant difference at baseline (baseline versus end of study). If both approaches were presented, the results from difference within difference were used. Where baseline values were not compared statistically, a visual inspection was carried out to assess equivalence. Sub-group analyses planned to focus on studies assessing the health setting, the CQI approach, key components of CQI that were previously identified as common across models, and assessed in studies (i.e., type and frequency of both training and meetings) and socio-economic health inequalities. The synthesis was presented as the number and proportion of studies in each group, with the narrative focusing on those RCTs finding no statistically significant difference between the CQI intervention and the comparator and those RCTs showing a statistically significant benefit from CQI in half or more of the outcomes assessed. This approach was used as the RCTs rarely identified their primary outcome measures, and it was felt that showing an effect on over half or more outcomes would limit the opportunity for selective reporting of specific outcomes where benefit was shown. Meta-analyses were not produced due to heterogeneity in the studies, particularly in the interventions and outcomes assessed.

**Table 2:** Categorisation of outcome measures

Proportion of outcomes in studies showing comparative benefit from CQI	Definition
No outcomes	No outcomes demonstrated a statistically significant difference between interventions in any study.
Under half of outcomes	Less than half of the outcomes in studies showed a statistically significant benefit from CQI versus its comparator.
Half of outcomes	Half of the outcomes in studies showed a statistically significant benefit from CQI versus its comparator.
More than half of outcomes	More than half of the outcomes in studies showed a statistically significant benefit from CQI versus its comparator.
All outcomes	All outcomes in the studies showed a statistically significant benefit from CQI versus its comparator.

## Results

**Fig. 1:**
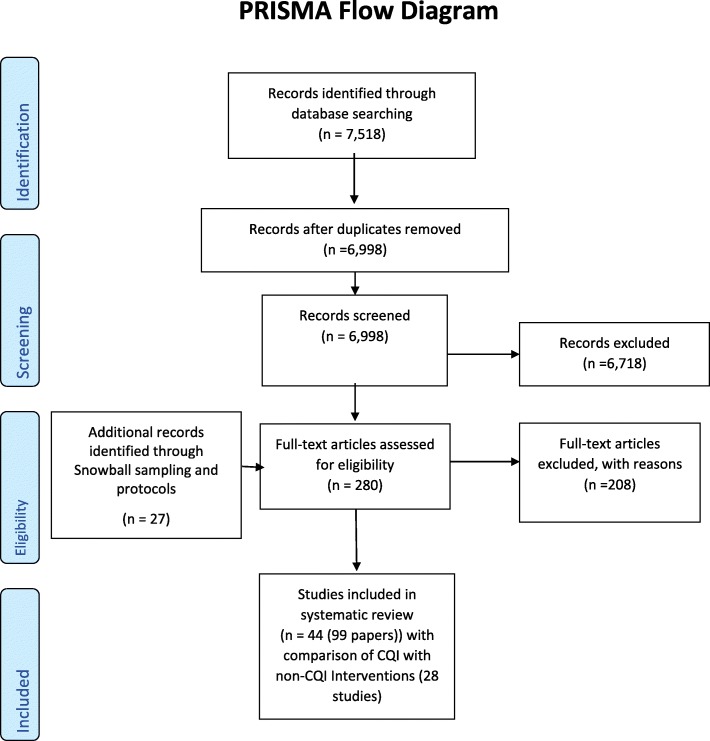
PRISMA Flow Diagram

Our search strategy identified 7518 papers which, after duplicate removal, resulted in 6998 papers for inspection. Screening of titles and abstracts excluded 6718 records (Fig. 1). Manuscripts for 280 papers were screened, with 44 studies presented in 72 papers included in the review. Some 27 additional link papers were identified through checking study protocols and snowball sampling. Although 44 RCTs met the selection criteria, the results presented are for 28 RCTs comparing CQI with other non-CQI interventions, whether considered current usual practice (i.e., usual care, normal practice, delayed intervention, or waiting list (19 RCTs)), a new management intervention without a CQI component (7 RCTs) or where no description was provided of the comparator (2 RCTs).

### Study characteristics

All 28 included studies were cluster RCTs. Most RCTs were carried out in high-income countries, with 15 in the USA [40–54], two in the Netherlands [55, 56], two in Canada [57, 58], two in the UK [59, 60], and one each in Sweden [61] and Spain [62]. Four RCTs were undertaken in the middle- or low-income countries, specifically in India [63], Mexico [64], Nigeria [65], and Malawi [66]. Another RCT was conducted across multiple African countries [67]. The clinical setting for the RCTs was mainly in primary (i.e., general practice) (13 RCTs) [40, 41, 43, 44, 48–53, 58, 62, 64] or secondary care (i.e., hospitals) (10 RCTs) [45, 54, 55, 59–61, 63, 66–68]. The remaining five RCTs were set in substance misuse clinics [69], community outreach [65], social services, and social care [47, 57] or tertiary care [56]. Most RCTs were published recently, with 19 RCTs published since 2010 [40, 41, 46–49, 51, 52, 54–58, 60, 63–67] and only 9 RCTs before 2010 [43–45, 50, 53, 59, 61, 62, 68]. The RCTs varied in the duration of the intervention, with 15 RCTs lasting 52 weeks or less [40, 43–45, 47–49, 52, 54, 57, 60–62, 65, 67], 11 RCTs more than 52 weeks [41, 42, 46, 51, 53, 55, 56, 58, 59, 64, 66]. Two RCTs used a stepped wedge design resulting in variation in intervention duration [50, 63]. Multi-disciplinary teams (MDT) were used in 19 RCTs [43–46, 49, 53, 55–61, 64–68, 70], with 8 RCTs not adequately describing membership of their teams [40, 47, 48, 50–52, 54, 63]. One RCT explicitly stated that they did not use an MDT approach [62]. PDSA was the CQI model most frequently used, with 12 RCTs using this approach [40, 43, 45, 46, 48, 50, 54, 57, 58, 63, 67, 70] and 7 RCTs using an adaptation of PDSA (the Model of Improvement (MoI)) [44, 55, 60, 61, 64–66]. One RCT used root cause analysis [47]. Eight RCTs used a range of undefined CQI approaches [49, 51–53, 56, 59, 62, 68].

Important characteristics of approaches to CQI were infrequently reported. Only 16 RCTs described the frequency of their team meetings, whether weekly (3 RCTs) [48, 49, 60], fortnightly (1 RCT) [44], monthly (10 RCTs) [41, 46, 47, 53, 54, 58, 59, 63, 65, 66] or quarterly or less frequently (2 RCTs) [55, 57]. The remaining 12 RCTs did not indicate the schedule of meetings [40, 43, 45, 50–52, 56, 61, 62, 64, 67, 68]. Duration of the meetings was rarely stated, with 7 RCTs reporting meetings that lasted either under 10 min [49], 40 to 70 min [48], 60 to 120 min [51, 53, 65], or 90 to 180 min [46, 57]. The other 21 RCTs did not describe duration of meetings [40, 43–45, 47, 50, 52, 54–56, 58–64, 66–68, 70]. The total number of meetings held also varied. Although 9 RCTs did not describe the number of meetings held [40, 45, 50, 61–64, 67, 68], 19 RCTs reported that they held either 1 to 4 [57], 5 to 9 [51, 54–56], 10 to 14 [43, 46, 52, 70], 15 to 20 [58], or more than 20 meetings [44, 47–49, 53, 59, 60, 65, 66]. Seventeen RCTs involved meetings that included organizational leaders as participants and discussed the implementation of the CQI [44, 46, 48, 49, 51, 53–55, 57–61, 63, 65, 66, 70]. In contrast, five RCTs involved organizational leaders in meetings but did not make it clear if the implementation of the CQI was discussed [40, 43, 47, 52, 56]. Six RCTs did not describe the nature of the meetings [45, 50, 64, 67, 68, 71]. 

Training, often thought fundamental to implementing CQI, was described in 24 RCTs [40, 44–54, 56, 57, 60–68, 70]. Fifteen RCTs used “in-person” training (i.e., meet for face to face training) [44, 46, 48–52, 54, 61, 62, 64–67, 70], eight RCTs used “in-person plus” training with the addition of other supporting elements (e.g., tele-/video-conferencing [40, 45], web-based materials [57, 60], handouts/manuals [53, 72] or combinations of support [56, 68]). One RCT used web-based training [47]. Duration of training ranged from 1–3 h [48, 56, 57, 64, 70], 4–8 h [49, 51], 9–16 h [45, 68], and over 16 h [44, 53, 60, 65]. Duration of training was not described in 15 RCTs [40, 43, 46, 47, 50, 52, 54, 55, 58, 59, 61–63, 66, 67].

### Risk of bias

**Fig. 2:**
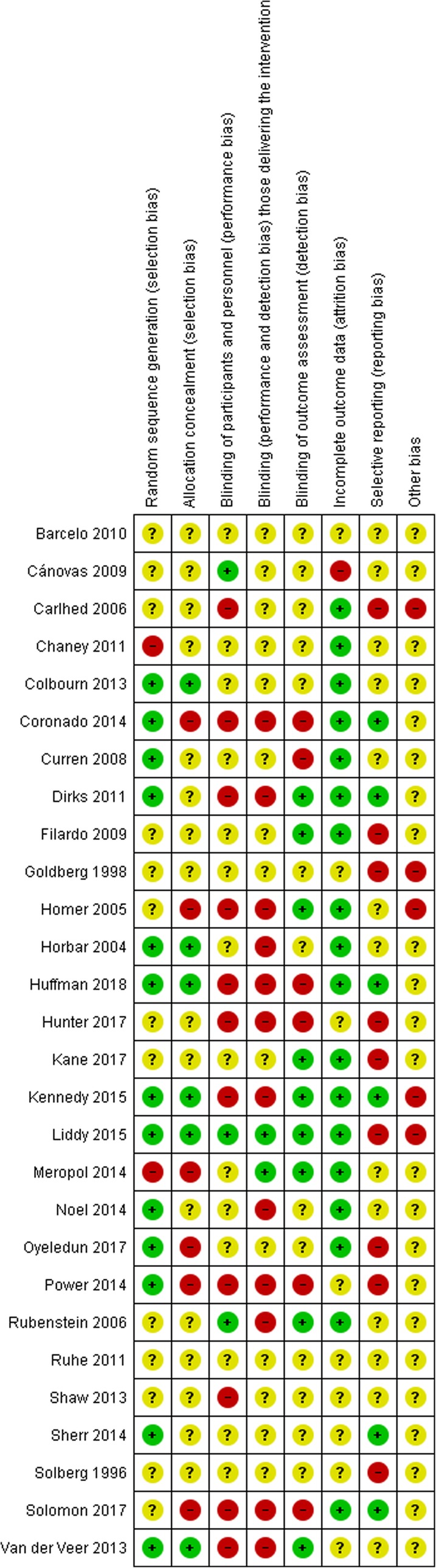
Risk of bias for included studies

Assessment of the risk of bias showed that the reliability of the results was uncertain due to the variability in the methodological rigor of the RCTs (Fig. 2). As such, findings should be interpreted with caution. Of the 28 RCTs, 26 RCTs had at least four criteria judged unclear or at high risk of bias [40, 43–56, 59–61, 63–68, 70, 71], with only 2 RCTs having five or more criteria judged low risk [57, 58].

### Effectiveness of CQI versus a non-CQI comparator

Of the 28 RCTs that compared CQI with a non-CQI intervention, 24 RCTs reported clinical process outcomes [40, 43–45, 48–55, 58, 60, 61, 63–68, 70, 71, 73], 17 RCTs reported patient outcomes [40, 43–47, 50, 54–59, 61, 63, 64, 66], and 3 RCTs reported other outcomes [46, 50, 65] (Tables 3, 4, and 5). The benefits that resulted from using CQI interventions over those provided by non-CQI comparators were limited. Over half of the RCTs reported no statistically significant difference between the interventions in their effect on any of the outcome measures assessed (clinical process 54.2% (13 RCTs) [43, 44, 50–52, 55, 58, 63–68]; patient 64.7% (11 RCTs) [40, 43, 44, 46, 55–59, 63, 66]; other 100% (3 RCTs) [46, 50, 65]). Improvements were reported. Some 29.2% of RCTs (7 RCTs [48, 49, 54, 57, 61, 62, 70]) assessing clinical process measures found a statistically significant comparative benefit from CQI on half or more of the outcomes. In contrast, 17.7% (3 RCTs [50, 61, 64]) and no RCTs found a beneficial effect on half or more of patient and other outcomes, respectively. The two RCTs at low risk of bias reported no difference between the interventions in terms of their effects on patient outcomes [57, 58]; however, one RCT showed a statistically significant benefit from the CQI intervention compared to non-CQI comparator on clinical process measures [57].

**Table 3:** RCTs evaluating the effects of CQI compared to non-CQI interventions on clinical process outcomes

Sub-group	Number of studies	Number (%) of RCTs reporting a statistically significant difference on different proportions of clinical process outcomes					
		All outcomes	Over half of outcomes	Half of outcomes	Under half of outcomes	No outcomes	One or more outcomes
All studies	24	4 (16.7%)	2 (8.3%)	1 (4.2%)	4 (16.7%)	13 (54.2%)	11 (45.8%)
Clinical background							
Primary Care	13	4 (30.8%)	0 (0%)	0 (0%)	2 (15.4%)	7 (53.8%)	6 (46.2%)
Secondary Care	9	0 (0%)	1 (11.1%)	1 (11.1%)	2 (22.2%)	5 (55.6%)	4 (44.4%)
Tertiary Care	0	0	0	0	0	0	0
Social Care	1	0 (0%)	1 (100.0%)	0 (0%)	0 (0%)	0 (0%)	1 (100.0%)
Other	1	0 (0%)	0 (0%)	0 (0%)	0 (0%)	1 (100.0%)	0 (0%)
Primary quality improvement model							
Plan-Do-Study-Act	11	2 (18.2%)	1 (9.1%)	1 (9.1%)	2 (18.2%)	5 (45.5%)	6 (54.5%)
Model for Improvement	7	0 (0%)	1 (14.3%)	0 (0%)	1 (14.3%)	5 (71.4%)	2 (28.6%)
Root cause analysis	0	0	0	0	0	0	0
Other	6	2 (33.3%)	0 (0%)	0 (0%)	1 (16.7%)	3 (50.0%)	3 (50.0%)
Training type							
Web-based	0	0	0	0	0	0	0
In person	14	4 (28.6%)	1 (7.1%)	1 (7.1%)	0 (0%)	8 (57.1%)	6 (42.9%)
In person plus	7	0 (0%)	1 (14.3%)	0 (0%)	4 (57.1%)	2 (28.6%)	5 (71.4%)
Not described	3	0 (0%)	0 (0%)	0 (0%)	0 (0%)	3 (100.0%)	0 (0%)
Training duration							
1-3 hours	4	2 (50.0%)	1 (25.0%)	0 (0%)	0 (0%)	1 (25.0%)	3 (75.0%)
4-8 hours	2	1 (50.0%)	0 (0%)	0 (0%)	0 (0%)	1 (50.0%)	1 (50.0%)
9-16 hours	2	0 (0%)	0 (0%)	0 (0%)	1 (50.0%)	1 (50.0%)	1 (50.0%)
>16 hours	4	0 (0%)	0 (0%)	0 (0%)	2 (50.0%)	2 (50.0%)	2 (50.0%)
Not described	12	1 (8.3%)	1 (8.3%)	1 (8.3%)	1 (8.3%)	8 (66.7%)	4 (33.3%)
Meetings							
Participant leader, implementation discussed	15	3 (20.0%)	2 (13.3%)	1 (6.7%)	2 (13.3%)	7 (46.7%)	8 (53.3%)
Participant leader, unclear implementation discussed	3	0 (0%)	0 (0%)	0 (0%)	1 (33.3%)	2 (66.7%)	1 (33.3%)
Not described	6	1 (16.7%)	0 (0%)	0 (0%)	1 (16.7%)	4 (66.7%)	2 (33.3%)
Meeting schedule							
Once a week or more	3	2 (66.7%)	0 (0%)	0 (0%)	1 (33.3%)	0 (0%)	3 (100.0%)
Fortnightly	1	0 (0%)	0 (0%)	0 (0%)	0 (0%)	1 (100.0%)	0 (0%)
Monthly	7	1 (14.3%)	0 (0%)	1 (14.3%)	1 (14.3%)	4 (57.1%)	3 (42.9%)
Quarterly or less frequent	2	0 (0%)	1 (50.0%)	0 (0%)	0 (0%)	1 (50.0%)	1 (50.0%)
Not described	11	1 (9.1%)	1 (9.1%)	0 (0%)	2 (18.2%)	7 (63.6%)	4 (36.4%)
Range of year of publication							
2010–2020	16	3 (18.8%)	1 (6.3%)	1 (6.3%)	2 (12.5%)	9 (56.3%)	7 (43.7%)
2000–2009	6	1 (16.7%)	1 (16.7%)	0 (0%)	1 (16.7%)	3 (50.0%)	3 (50.0%)
1990–1999	2	0 (0%)	0 (0%)	0 (0%)	1 (50.0%)	1 (50.0%)	1 (50.0%)

**Table 4:** RCTs evaluating the effects of CQI compared to non-CQI Interventions on patient outcome measures

Subgroup	Number of studies	Number (%) of RCTs reporting a statistically significant difference on different proportions of patient outcomes					
		All outcomes	Over half of outcomes	Half of outcomes	Under half of outcomes	No outcomes	One or more outcomes
All studies	17	0 (0%)	2 (11.8%)	1 (5.9%)	3 (17.6%)	11 (64.7%)	6 (35.3%)
Clinical background							
Primary Care	6	0 (0%)	1 (16.7%)	1 (16.7%)	0 (0%)	4 (66.7%)	2 (33.3%)
Secondary Care	7	0 (0%)	1 (14.3%)	0 (0%)	2 (28.6%)	4 (57.1%)	3 (42.9%)
Tertiary Care	1	0 (0%)	0 (0%)	0 (0%)	0 (0%)	1 (100.0%)	0 (0%)
Social Care	2	0 (0%)	0 (0%)	0 (0%)	1 (50.0%)	1 (50.0%)	1 (50.0%)
Other	1	0 (0%)	0 (0%)	0 (0%)	0 (0%)	1 (100.0%)	0 (0%)
Primary quality improvement model							
Plan-Do-Study-Act	9	0 (0%)	0 (0%)	1 (11.1%)	2 (22.2%)	6 (66.7%)	3 (33.3%)
Model for Improvement	5	0 (0%)	2 (40.0%)	0 (0%)	0 (0%)	3 (60.0%)	2 (40.0%)
Root cause analysis	1	0 (0%)	0 (0%)	0 (0%)	1 (100.0%)	0 (0%)	1 (100.0%)
Other	2	0 (0%)	0 (0%)	0 (0%)	0 (0%)	2 (100.0%)	0 (0%)
Training type							
Web-based	1	0 (0%)	0 (0%)	0 (0%)	1 (100.0%)	0 (0%)	1 (100.0%)
In person	7	0 (0%)	2 (28.6%)	1 (14.3%)	1 (14.3%)	3 (42.9%)	4 (57.1%)
In person plus	5	0 (0%)	0 (0%)	0 (0%)	1 (20.0%)	4 (80.0%)	1 (20.0%)
Not described	4	0 (0%)	0 (0%)	0 (0%)	0 (0%)	4 (100.0%)	0 (0%)
Training duration							
1–3 hours	3	0 (0%)	1 (33.3%)	0 (0%)	0 (0%)	2 (66.7%)	1 (33.3%)
4–8 hours	0	0	0	0	0	0	0
9–16 hours	1	0 (0%)	0 (0%)	0 (0%)	1 (100.0%)	0 (0%)	1 (100.0%)
>16 hours	1	0 (0%)	0 (0%)	0 (0%)	0 (0%)	1 (100%)	0 (0%)
Not described	12	0 (0%)	1 (8.3%)	1 (8.3%)	2 (16.7%)	8 (66.7%)	4 (33.3%)
Meetings							
Participant leader, implementation discussed	10	0 (0%)	1 (10.0%)	0 (0%)	1 (10.0%)	8 (80.0%)	2 (20.0%)
Participant leader, unclear implementation discussed	4	0 (0%)	0 (0%)	0 (0%)	1 (25.0%)	3 (75.0%)	1 (25.0%)
Not described	3	1 (33.3%)	1 (33.3%)	0 (0%)	1 (33.3%)	0 (0%)	3 (100.0%)
Meeting schedule							
Once a week or more	0	0	0	0	0	0	0
Fortnightly	1	0 (0%)	0 (0%)	0 (0%)	0 (0%)	1 (100.0%)	0 (0%)
Monthly	7	0 (0%)	0 (0%)	0 (0%)	2 (28.6%)	5 (71.4%)	2 (28.6%)
Quarterly or less frequent	2	0 (0%)	0 (0%)	0 (0%)	0 (0%)	2 (100.0%)	0 (0%)
Not described	7	0 (0%)	2 (28.6%)	1 (14.3%)	1 (14.3%)	3 (42.9%)	4 (57.1%)
Range of year of publication							
2010–2020	11	0 (0%)	1 (9.1%)	0 (0%)	2 (18.2%)	8 (72.7%)	3 (27.3%)
2000–2009	5	0 (0%)	1 (20.0%)	1 (20.0%)	1 (20.0%)	2 (40.0%)	3 (60.0%)
1990–1999	1	0 (0%)	0 (0%)	0 (0%)	0 (0%)	1 (100.0%)	0 (0%)

**Table 5:** RCTs evaluating the effects of CQI compared to non-CQI interventions on other outcome measures

Subgroup	Number of studies	Number (%) of RCTs reporting a statistically significant difference on different proportions of other outcomes					
		All outcomes	Over half of outcomes	Half of outcomes	Under half of outcomes	No outcomes	One or more outcomes
All studies	3	0 (0%)	0 (0%)	0 (0%)	0 (0%)	3 (100.0%)	0 (0%)
Clinical background							
Primary Care	1	0 (0%)	0 (0%)	0 (0%)	0 (0%)	1 (100.0%)	0 (0%)
Secondary Care	0	0	0	0	0	0	0
Tertiary Care	0	0	0	0	0	0	0
Social Care	0	0	0	0	0	0	0
Other	2	0 (0%)	0 (0%)	0 (0%)	0 (0%)	2 (100.0%)	0 (0%)
Primary quality improvement model							
Plan-Do-Study-Act	2	0 (0%)	0 (0%)	0 (0%)	0 (0%)	2 (100.0%)	0 (0%)
Model for Improvement	1	0 (0%)	0 (0%)	0 (0%)	0 (0%)	1 (100.0%)	0 (0%)
Root cause analysis	0	0	0	0	0	0	0
Other	0	0	0	0	0	0	0
Training type							
Web-based	0	0	0	0	0	0	0
In person	3	0 (0%)	0 (0%)	0 (0%)	0 (0%)	3 (100.0%)	0 (0%)
In person plus	0	0	0	0	0	0	0
Not described	0	0	0	0	0	0	0
Training duration							
1–3 hours	0	0	0	0	0	0	0
4–8 hours	0	0	0	0	0	0	0
9–16 hours	0	0	0	0	0	0	0
>16 hours	1	0 (0%)	0 (0%)	0 (0%)	0 (0%)	1 (100.0%)	0 (0%)
Not described	2	0 (0%)	0 (0%)	0 (0%)	0 (0%)	2 (100.0%)	0 (0%)
Meetings							
Participant leader, implementation discussed	2	0 (0%)	0 (0%)	0 (0%)	0 (0%)	2 (100.0%)	0 (0%)
Participant leader, unclear implementation discussed	0	0	0	0	0	0	0
Not described	1	0 (0%)	0 (0%)	0 (0%)	0 (0%)	1 (100.0%)	0 (0%)
Meeting schedule							
Once a week or more	0	0	0	0	0	0	0
Fortnightly	0	0	0	0	0	0	0
Monthly	2	0 (0%)	0 (0%)	0 (0%)	0 (0%)	2 (100.0%)	0 (0%)
Quarterly or less frequent	0	0	0	0	0	0	0
Not described	1	0 (0%)	0 (0%)	0 (0%)	0 (0%)	1 (100.0%)	0 (0%)
Range of year of publication							
2010–2020	2	0 (0%)	0 (0%)	0 (0%)	0 (0%)	2 (100.0%)	0 (0%)
2000–2009	1	0 (0%)	0 (0%)	0 (0%)	0 (0%)	1 (100.0%)	0 (0%)
1990–1999	0	0	0	0	0	0	0

### Sub-group analyses

Findings were similar in the sub-group analyses that investigated the influence of the health setting, type of CQI model used, and the influence of specific core features of the CQI approach (e.g., type and duration of training, type and schedule of meetings). In most sub-groups, over 50% of RCTs reported no statistically significant benefit from CQI compared to the non-CQI comparator on all the outcomes assessed. For the outcomes defined as “other,” this included all three RCTs finding no statistically significant effect [46, 50, 65]. There were some exceptions where more than 50% of RCTs reported a statistically significant benefit from CQI. These were limited to the effects of specific types of training (i.e., clinical process outcomes: in-person plus training; patient outcomes: in-person training), as well as types and frequencies of meetings (i.e., patient outcomes: not described) on the clinical process and patient outcomes. Benefits from the use of CQI compared to non-CQI comparators were evident, although these varied between the different sub-groups and outcomes considered.

### Care setting

In terms of the setting of care, CQI appeared marginally more effective in primary care than in secondary care. Over 30% of RCTs in primary care reported a statistically significant improvement in half or more of the clinical process (4 RCTs) [48, 49, 70, 71] and patient outcomes (2 RCTs) [50, 64] compared to less than 23% for secondary care for clinical process (2 RCTs) [54, 61] and patient outcomes (1 RCT) [61]. The effectiveness of CQI in other settings (i.e., tertiary care, social care, or other) was less clear given the limited evidence available [46, 47, 56, 57, 65].

### CQI models

PDSA and the MoI were the main CQI models used. Although PDSA appeared more effective than MoI in improving half or more of clinical process outcomes in RCTs (36.4% (4 RCTs) [48, 54, 57, 70] versus 14.3% (1 RCT) [61], respectively), the reverse was found for patient outcomes (11.1% (1 RCT) [50] versus 40% (2 RCTs) [61, 64], respectively). Other unspecified models of CQI also appeared effective in impacting on half or more of the clinical process outcomes in 33.3% of RCTs (2 RCTs) [49, 71].

### Training type and duration

In-person training was used most frequently and had the largest influence on outcomes, leading to statistically significant improvements in half or more of outcomes in 42.8% (6 RCTs) [48, 49, 54, 61, 70, 71] and 42.9% (3 RCTs) [50, 61, 64] of RCTs assessing clinical process and patient outcomes respectively. Person plus training with additional elements was beneficial in half or more of outcomes in 14.3% (1 RCT) [57] of RCTs assessing clinical process outcomes. Although a range of training durations were used, shorter training durations appeared more effective. Training sessions of 1 to 3 h and 4 to 8 h were beneficial in improving half or more outcomes in 75% (3 RCTs [48, 57, 70] and 50% (1 RCT [64], respectively) of RCTs assessing clinical process outcomes. Similarly, training lasting 1 to 3 h was shown to be beneficial for 33.3% (1 RCT) [64] of RCTs assessing patient outcomes. Training where the duration was not described had some beneficial effects on half or more of outcomes in 24.9% (3 RCTs) [54, 61, 71] and 16.6% (2 RCTs) [50, 61] of RCTs assessing clinical process and patient outcomes, respectively.

### Meeting type and frequency

The type of meeting and their frequency appear to have some influence on the effectiveness of CQI. When it was clear that meetings involved a discussion of the implementation of the improvement initiatives, a higher proportion of RCTs (40% (6 RCTs)) [48, 49, 54, 57, 61, 70] found a statistically significant benefit on half or more of the clinical process outcomes reported compared to when it was not discussed (0%). Where patient outcomes were assessed, meetings that were not described had a statistically significant beneficial effect on half or more outcomes in more RCTs than other types of meetings (66.6% (2 RCTs)) [50, 64]. The effects of meeting frequency were less clear. Meetings that were at least weekly (66.7% (2 RCTs)) [48, 49] and meetings that were monthly (28.6% (2 RCTs)) [54, 70], appeared to be more effective than other meeting schedules in producing statistically significant improvements in half or more outcomes in RCTs assessing clinical process measures. In contrast, meetings that did not describe their frequency had greater influence on RCTs reporting patient outcomes (42.9% (3 RCTs)) [50, 61, 64].

### Range of year of publication

The majority of RCTs were published from 2010 to 2020. There appeared to be no consistent improvement in the effectiveness of CQI over time for all outcomes. Similar effects were reported when RCTs published between 2000 and 2009 (33.3% (2 RCTs)) [61, 71] were compared with those between 2010 and 2020 (31.4% (5 RCTs)) [48, 49, 54, 57, 70] in producing statistically significant improvements in half or more clinical process outcomes. For patient outcomes, a difference was evident with fewer RCTs reporting a statistically significant improvement in half or more outcomes between 2010 and 2020 (9.1% (1 RCT)) [50] than 2000 and 2009 (40% (2 RCTs)) [50, 61].

## Discussion

Increasingly the provision of health and social care has been shaped by the challenges of a growing demand for services, pressures on available funding and a continued drive for efficiency [1]. Different approaches have been adopted in an attempt to maintain the comprehensiveness and quality of care, and to tackle inequity in provision of services [74]. Recently, attention has shifted to improving services by developing the capabilities and capacity of organizations through building their knowledge, skills, and infrastructure [74]. The focus on system-level quality improvement has resulted in CQI methods being identified, and increasingly used, as an approach to enhance the quality of care and reduce costs [3–9, 72]. Despite its effectiveness within industrial and manufacturing sectors, it remains unclear whether CQI could be successfully employed in the health care sector. In systematically reviewing the evidence comparing the use of CQI with non-CQI interventions in health care, it was apparent that, regardless of the growth in evidence in the last 10 years, the results were largely equivocal. Although this appears to perpetuate much of the uncertainty, we identified elements of CQI that may prove beneficial in improving outcomes and possible reasons for our findings that may inform further research.

Our findings appear to concur with those of previous systematic reviews on developing professional practice and improving health care outcomes [26, 31], whether showing limited benefit [31], the influence of different components [26, 31], and/or reasons for the continuing uncertainties [22, 26, 28, 31, 32]. Where CQI appeared effective, collaboration and communication between health care professionals appeared important. We found that meetings helped to facilitate the implementation of CQI, particularly when meetings were led by participant leaders, who were an integral part of multidisciplinary teams, focusing implementation of initiatives through cooperative working. If these meetings were held frequently, such as weekly rather than monthly, this seemed to improve the effectiveness of the CQI approach taken. The importance of direct communication was re-enforced through the benefits reported for CQI initiatives that used person focused face to face training, which appeared more effective than other forms of training (e.g., web-based training or combinations of training methods) and were thought to help improve competence and motivation [75]. Others have found similar effects through different forms of interaction between those involved in CQI [28, 30, 76]. Audit and feedback have been recognized as important facilitators when implementing CQI, with increased intensity of support more effective in helping to incorporate improvements into practice [28, 30, 76]. The impact of collaboration and active communication may help to explain the apparent benefits from the use of CQI in primary care, where team structures reflect those used in operationalizing CQI methods [77, 78] and such initiatives are incentivized through other mechanisms (e.g., Quality and Outcomes Framework) [79]. Despite several different approaches to CQI, we identified that PDSA and MoI were the models most frequently used, showing benefit on clinical process and patient outcomes in a third of trials respectively. PDSA was previously reported to be an effective approach in improving health outcomes [32]. The rationale for the use of PDSA and MoI, and the reasons for their effectiveness in specific situations, has proven difficult to clarify. This may reflect the frequent adaptation of CQI models during implementation rendering the differences unclear [80], that models often have overlapping features [17] and frequently there is incomplete or inconsistent reporting of the details of the approach taken [22, 26, 32]. Although the evidence base has grown in recent years, there has been no discernible change in the effectiveness of CQI within the health care setting. This may be due to several factors; however, its likely to reflect the fact that studies undertaken are heterogeneous in nature through the approaches to CQI used, populations studied, and outcomes reported. Socio-economic health inequalities were not reported in any RCTs, which is not uncommon outside public health research, appearing to reflect their primary focus on the health condition and not the other underlying determinants of population health.

The limited effects of CQI initiatives may reflect several factors. First, health and social care organizations, both nationally and locally, are complex organizations which may lack the necessary structure, resources, and resolve to operationalize CQI initiatives effectively and consistently [81, 82]. Given the opportunity for approaches to CQI to be adapted to local conditions, there is a chance for variation in their implementation. This may reduce the inherent strengths of the CQI approach, limit its effectiveness and make it more difficult to research. Second, CQI initiatives are often implemented over a short period, restricting the opportunity to affect the different outcome measures assessed in the RCTs, particularly patient-based outcomes. Third, recognition of the importance of different components used in CQI (e.g., audit, feedback, meetings, and training), has resulted in their adoption as part of standard management practice. Consequently, they are increasingly part of different management interventions that are compared in trials, effectively controlling for their effects. Fourth, identifying the reasons underlying the effectiveness of specific approaches to CQI has proven difficult to clarify. This may reflect their frequent adaptation during implementation and that details of the approach were often incompletely reported [22, 26, 32]. Although a pragmatic approach to the use of CQI may be necessary in practice, adherence to the core components and more complete reporting of the different models used in trials would help to distinguish which models and elements are most effective [22, 26, 28, 31, 32]. Fifth, the limited evidence identified and its poor quality may result in uncertainty in the findings. The unclear or high risk of bias reported for most RCTs may reflect either the inherent challenges in conducting RCTs of CQI initiatives (e.g., blinding in cluster RCTs) or a lack of understanding of the importance of ensuring, and reporting, the rigor used in implementing the study methodology. It may be that the use of RCTs for evaluating CQI is undermined by the challenges faced and other approaches could compliment such experimental studies [82].

The systematic review had certain strengths, including the following: it was produced following a registered research protocol by independent researchers, clearly describing the methods followed; identified evidence through comprehensive searches of electronic databases, reference checking and citation checks; selected studies, extracted data, and assessed risk of bias using standard pre-piloted forms and processes; and involved public advisors in commenting on the research protocol and final report. Also, it had certain limitations, such as searches could have been extended to other sources; inclusion criteria were limited to RCTs which, although the gold standard for assessing effectiveness through limiting potential confounding, may restrict the opportunity to assess more real-world evidence provided by other comparative study designs; comparisons were with non-CQI approaches, removing the opportunity to directly compare between different CQI approaches; details of the studies were limited in the publications and further evidence was not obtained from study authors; extraction of data and assessment of risk of bias were undertaken by a single reviewer with information checked by a second reviewer, providing the opportunity for error; the synthesis categorized the evidence, limiting the extent of data presented from each RCT; and a meta-analysis was not undertaken.

Further research into the effectiveness of CQI interventions in health and social care would be beneficial. A systematic review comparing different CQI models and other active comparisons may help to identify the elements of these approaches that are useful to organizations. It could include experimental and non-experimental comparative studies and look at the specific influence of potentially important moderators (e.g., training methods/type and focus of meeting). If further RCTs are going to be undertaken it is important that they take a mixed-method approach, as it is currently unclear within the literature exactly which moderators are important. Any RCTs should be conducted by independent researchers that assess outcomes over a longer period, as this would help to clarify if the benefits could be realized in terms of clinical process or organizational outcomes and, more importantly, for patient-related outcomes. The RCTs could specifically compare the different key components that have been identified as core to the different approaches to CQI. Any RCT that is undertaken should report against a standard set of outcomes, provide full descriptions of all elements of the CQI process, and consider health inequalities. It has been evident that the quality of the evidence and the quality of its reporting is poor, preventing a full understanding of the findings and the context in which they have been attained. This should be addressed.

## Conclusion

CQI is an important and proven approach to improving the quality and efficiency of industrial processes, which has drawn considerable and growing attention in health care. Evaluations of its use in health have been inadequate, causing uncertainty as to its benefits. It is evident that in certain situations, it has had significant effects on improving the provision of health care, although these were limited. Further independent research is required to clarify what approaches to CQI may be employed to improve the quality and efficiency of service provision.

## Supplementary information

**Additional file 1.** PRISMA 2009 Checklist

**Additional file 2.** Search strategy used for MEDLINE (via Ovid) from database inception to 23 February 2019

## Data Availability

Data sharing is not applicable to this article as no datasets were generated or analyzed during the current study. All original data synthesised can be obtained from the selected studies.

## Abbreviations

AMED: Allied and Complimentary Medicine Database
CINAHL: Current Nursing and Allied Health Literature
CQI: Continuous quality improvement
HMIC: Healthcare Management Information Consortium
LISTA: Library Information Science and Technology Abstracts
MDT: Multi-disciplinary team
MOI: Model of Improvement
n: Number
NHS EED: NHS Economic Evaluation Database
PDSA: Plan, Do, Study, Act
PRISMA: Preferred Reporting Items for Systematic Reviews and Meta-Analyses
RCT: Randomized controlled trial
