# Supplementary material for: The effectiveness of continuous quality improvement for developing professional practice and improving health care outcomes: a systematic review
Source: Implement Sci. 2020 Apr 19;15:23. doi: 10.1186/s13012-020-0975-2 (PMC7168964; doi:10.1186/s13012-020-0975-2)
Supplement: Supplementary file 2 — Additional file 2. Search strategy used for MEDLINE (via Ovid) from database inception to 23 February 2019 [file 13012_2020_975_MOESM2_ESM.docx]

Additional File 2: Search strategy used for MEDLINE (via Ovid) from database inception to 23^rd^ February 2019

| 1. (quality adj2 ((continuous$ or total) adj2 (manag$ or improv$))).tw. |
| --- |
| 1. ((continuous$ or total) adj1 (quality adj1 (manag$ or improv$))).tw. |
| 1. (CQI or TQM).tw. |
| 1. total quality management/ |
| 1. quality manag$.tw. |
| 1. ((process or processes or system or systems) adj1 (improving or improvement or improve or redesign$)).tw. |
| 1. ((improvement or QI or quality assurance or QA) adj3 (team? or microsystem? or cycle?)).tw. |
| 1. (PDSA or PCDA or TQIS or plan do study or plan do check).tw. |
| 1. ((shewhart or shewart or deming) adj3 (cycle or method)).tw. |
| 1. rapid cycle.tw. |
| 1. (quality adj2 collaborative?).tw. |
| 1. (breakthrough adj3 (series or project or collaborative?)).tw. |
| 1. (lean adj1 (approach or management or method? or methodology or thinking or enterpri#e or practice or philosophy or principles)).tw. |
| 1. six sigma.tw. |
| 1. randomized controlled trial.pt. |
| 1. random$.tw. |
| 1. control$.tw. |
| 1. intervention$.tw |
| 1. evaluat$.tw. |
| 1. or/15-19 |
| 1. animal/ |
| 1. human/ |
| 1. 21 not (21 and 22) |
| 1. 22 not 23 |
| 1. Taguchi.mp |
| 1. Plan-Do-Study-Act.mp. |
| 1. "Plan-Do-Check-Act".mp. |
| 1. business process reengineering.mp. |
| 1. "define-measure-analyse-improve-control".mp. |
| 1. "define-measure-analyse-design-verify".mp. |
| 1. iterative cycle.mp. |
| 1. Kansei.mp. |
| 1. Kaizen.mp. |
| 1. "quality function deployment".mp. |
| 1. "House of quality".mp. |
| 1. "Toyota production system".mp. |
| 1. "lean manufacturing".mp. |
| 1. "continuous quality improvement".mp. |
| 1. "root cause".ab,ti. |
| 1. "value stream".ab,ti. |
| 1. practice change.ab,ti. |
| 1. (organisational change or organizational change).mp. |
| 1. team quality improvement sequence.mp. |
| 1. model for improvement.mp |
| 1. statistical process control.mp. |
| 1. model for improvement.tw. |
| 1. 1 or 2 or 3 or 4 or 5 or 6 or 7 or 8 or 9 or 10 or 11 or 12 or 13 or 14 or 25 or 26 or 27 or 28 or 29 or 30 or 31 or 32 or 33 or 34 or 35 or 36 or 37 or 38 or 39 or 40 or 41 or 42 or 43 or 44 or 45 or 46 |
| 1. 20 and 24 and 47 |
